# Sustained Gaze Is a Reliable In-home Test of Attention for Aging Pet Dogs

**DOI:** 10.3389/fvets.2021.819135

**Published:** 2021-12-23

**Authors:** Jane A. Hoel, Ginger B. Templeton, Gilad Fefer, Beth C. Case, Anshu Shah, Margaret E. Gruen, Natasha J. Olby

**Affiliations:** ^1^Department of Clinical Sciences, College of Veterinary Medicine, North Carolina State University, Raleigh, NC, United States; ^2^College of Veterinary Medicine, Michigan State University, East Lansing, MI, United States; ^3^Comparative Medicine Institute, North Carolina State University, Raleigh, NC, United States

**Keywords:** Canine Cognitive Dysfunction Syndrome, cognitive testing, dementia, executive function, attention, cognitive impairment

## Abstract

Canine Cognitive Dysfunction Syndrome (CCDS) is a syndrome of progressive cognitive decline comparable to Alzheimer's Disease. The sustained gaze test captures attention loss associated with CCDS in laboratory settings, and adapting the sustained gaze test for use by owners at home could greatly increase the data generated on CCDS. We hypothesized that it would be feasible for owners to perform the sustained gaze test at home, and that results would be reliable over repeated trials. Training materials were developed and dog owners underwent training and performed the test in triplicate at weekly intervals for 3 weeks. Gaze videos and a CAnine DEmentia Scale (CADES) questionnaire were submitted each week. Videos were examined for inclusion and duration of gaze was recorded. One observer repeated video assessments twice, 1 week apart; five different observers assessed videos once. Outcome measures included the relationship between CADES and gaze duration, test-retest reliability of owner-performed sustained gaze testing, and intra- and inter-rater reliability. Twenty dogs aged 7–15.5 years completed testing. The majority of videos were acceptable (162/183). Within dog test-retest reliability was excellent (ICC = 0.96). Intra- and interobserver reliability for determining video validity for inclusion were substantial (*k* = 0.76 and 0.78, respectively); for duration of gaze these were excellent (ICC = 0.99 and 0.96, respectively). Gaze duration was significantly associated with CADES (*p* = 0.0026). We conclude that owners can perform the sustained gaze test at home and that data generated are reliable and correlate to CADES, a validated measure of dementia.

## Introduction

Canine Cognitive Dysfunction Syndrome (CCDS) is a syndrome of progressive deterioration in cognition associated with amyloid deposition and cortical atrophy and it is considered the canine analog to Alzheimer's Disease in humans (1–14). The symptoms associated with CCDS fall into the following domains: disorientation, social interaction changes, sleep/wake cycle alterations, house soiling, activity changes, anxiety, and deficits in learning and memory (4, 5, 8–10, 15). The prevalence of CCDS is high in aging dogs. One study estimated a prevalence of 14.2–22.5% in animals 8 years and older (4), while another reported that 23% of dogs 11–12 years old and 68% of dogs 15–16 years old had at least 1 sign consistent with CCDS (15). Several studies have reported a dramatic increase in prevalence associated with increased age of dogs (7, 10, 15). However, while there are numerous studies documenting owner reported signs, studies aimed at quantifying behavioral and cognitive changes using specific validated testing are currently lacking (12, 16, 17).

There are numerous potential causes for cognitive decline in aging dogs including systemic disease such as liver disease, and intracranial disease such as brain neoplasia. In order to establish a diagnosis of CCDS, dogs should have a consistent history, normal physical and neurological examination, normal blood work, and cortical atrophy on MRI. Unfortunately, the cost and risk of performing advanced imaging under general anesthesia in aging dogs means that there is heavy reliance on physical examination findings and clinical metrology instruments (2, 18). The CAnine DEmentia Scale (CADES) is one such validated instrument that effectively identifies three stages of cognitive impairment: mild, moderate, and severe (2, 19). We have found that CADES scores and impairment categories correlate with markers of neuronal death and amyloid pathology and to behavioral tests of attention (13, 14).

Cognitive function can be evaluated in a laboratory setting in pet dogs (3, 12, 14, 16, 17, 20–22). Various different domains are assessed including executive function, social cues, and working memory. Previous studies in both humans and dogs have documented that sustained attention and social interaction decline with age and onset of dementia (17, 20). We have found that the duration of a sustained gaze test, adapted from an assessment of social contact/empathy (23) inversely correlates with CADES score and with plasma concentrations of neurofilament light chain (14) and amyloid beta 40 and 42 (13). We therefore propose that it is a powerful and simple method of tracking cognitive decline in pet dogs (12).

While the sustained gaze test has been validated for in-laboratory use, the validity and test-retest reliability of owner-performed sustained gaze testing in the home is unknown. Performance at home has been evaluated for a version of the sustained gaze test when completed as part of a larger cognitive battery, and was found to be feasible (23) but was only measured at one timepoint. The ability to quantify pet dogs' attention span by owners at home could facilitate data collection in longitudinal studies and increase case recruitment in clinical trials of canine aging. We hypothesize that owners can perform the sustained gaze test at home with their dogs and produce reliable data. The aims of this study were to develop training modules for at home sustained gaze testing, to identify barriers to performing the sustained gaze test in the home, to evaluate test-retest reliability of owner-performed sustained gaze testing as well as intra- and inter-rater reliability for the inclusion of a test trial and duration of gaze.

## Methods

### Training Module and Questionnaire Development

**Figure 1:**
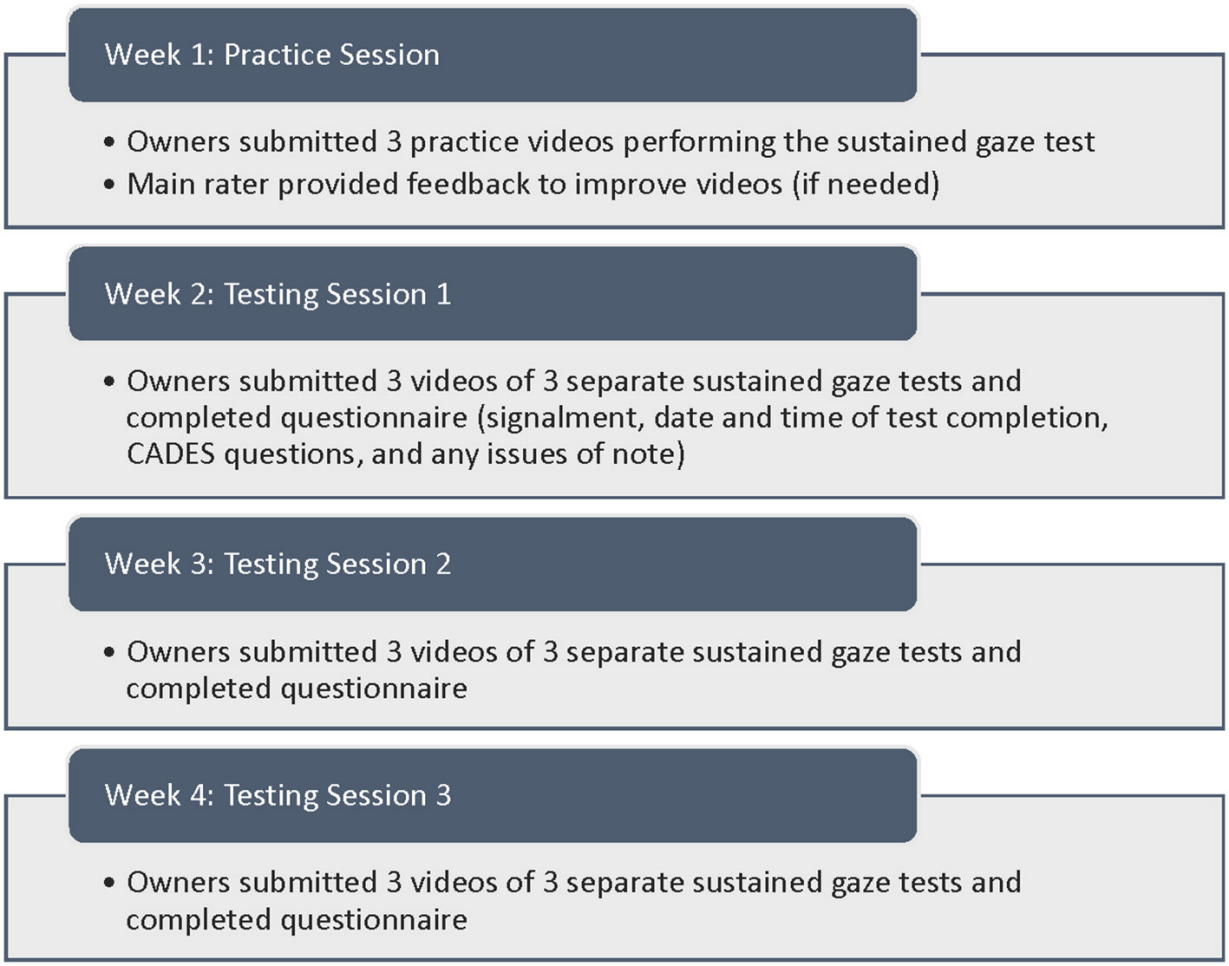
Chart depicting the testing performed each week of the study.

A training module was developed to teach owners how to perform the sustained gaze test in the home environment and upload video submissions. The first training video described the sustained gaze test and showed examples of the test being performed, both in the laboratory and home environments (https://youtu.be/yMFUtQ-9qz0). The video also outlined the testing timeline (Figure 1), suggestions for success, and behaviors that result in test exclusion. A second training video provided instructions for uploading videos from both mobile devices and computers to our secure Google form. Both videos were deployed via our own YouTube channel. All owners reviewed and signed an informed consent. Owners uploaded the videos of their dogs' tests on each testing occasion. A questionnaire was created for owners to fill out each week, corresponding to each round of sustained gaze testing. Owners provided their dog's signalment, time of day the tests were completed, and if there were any testing issues of note. The behavioral questionnaire also included the CAnine DEmentia Scale (CADES) to allow comparison of sustained gaze testing to owner quantification of dementia (2) (Supplementary Material 1). Scores from CADES were categorized into normal (score 0–7), mild (8–23), moderate (24–44), and severe (>45) cognitive impairment. After initial deployment of the training module, instructions were modified prior to performing the weekly testing protocol to address common problems with the testing performed by owners. All owners reviewed the updated instructions prior to performing the test-retest experiment. All protocols were reviewed and approved by the NCSU Institutional Animal Care and Use Committee.

### Sustained Gaze Test

Instructions for performing the sustained gaze test were as follows: using a smartphone, start the video recording and call the dog over, show the treat to the dog while bringing the treat up beside the owner's face, capture the dog's face in the video frame, remain quiet, and continue recording until a few seconds after the dog breaks eye contact (looking away from the owner, phone, or treat), ending the gaze. Since the upper limit of the test is 60 s, owners were instructed to stop recording if their dog maintained a gaze longer than 60 s without breaking eye contact. Owners were instructed to refrain from immediately rewarding their dog with the treat after they break their gaze to prevent training dogs to look away from the owner and/or treat. A short break was taken to regroup between repetitions, the duration of which was dictated by the individual dog's needs. To minimize confounding variables, owners were asked to adhere to a few guidelines. Testing should be performed in a quiet room, to avoid disturbance by other family members or pets; the test should be performed on a carpeted or non-slip surface, because aging dogs may have trouble maintaining postures on slippery floors; a high value treat should be used with testing at least 2 h after eating to ensure food motivation; and finally, the same person should always do the testing and testing should be performed at the same time of day. Before beginning testing, owners submitted a practice video to ensure proper technique and underwent additional training if needed. Once trained, owners performed 3 sustained gaze tests per session, doing a single session per week for a total of 3 weeks of testing.

### Animals

Dogs and owners were recruited from the faculty, staff, and students of the North Carolina State University College of Veterinary Medicine. To meet inclusion criteria, participating dogs were required to be systemically healthy, in the senior or geriatric age bracket, and appropriately food motivated. Owners had to be able to videotape and upload videos of sustained gaze testing. We defined senior dogs to be in the last 25% of the average lifespan (24) of their breed and geriatric dogs to be at or beyond their average breed lifespan, as published by the American Kennel Club (25). All procedures were approved by the North Carolina State University Institutional Animal Care and Use Committee.

### Video Analysis

Errors in testing methods observed in the initial training videos and owner comments from the initial forms were used to improve the training instruction. Videos from the test-retest experiment were viewed initially to look for exclusionary behaviors by both dogs and owners and recorded as pass or fail. Videos were determined to be valid for inclusion if the beginning and end of the gaze were visible and no exclusionary factors were present (such as overt distractions, scratching, sneezing, or calling the dog's name). Gaze times for each video were recorded in seconds, rounded to the nearest whole number, and were capped at 60 s. The mean gaze time (seconds) was calculated from the three videos submitted at each of the dog's weekly sessions and these values were used for assessment of test-retest reliability.

Intra- and inter-rater reliability for determining pass/fail validity of videos and gaze timing were also assessed. Ten randomly chosen videos were reviewed by one rater to determine pass/fail validity of the testing and record gaze times on two separate occasions, 1 week apart. Previously recorded data were masked from the rater by entering data on a new spreadsheet. Five separate raters reviewed 23 distinct videos selected to include a wide range of behaviors, gaze times, and settings. After training raters on video analysis and exclusion criteria, raters assessed pass/fail validity, and gaze times for each of the 23 videos. Raters were blinded to identifying information.

### Statistical Analysis

Summary data were generated for the dogs participating in the study including age, breed, sex, CADES scores and categories, and mean gaze time for each testing session. The mean of the weekly CADES scores for each dog was calculated and used to determine the overall corresponding CADES category for each dog, provided in Table 1. Test-retest reliability of the sustained gaze test and CADES score and category was determined using the intraclass correlation coefficient (ICC) for continuous data and kappa analysis for categorical data. The relationship between the sustained gaze test, CADES score and category, and age were examined using logistic regression. Intra- and inter-rater reliability for the gaze time measurements were determined using ICC. Intra- and inter-rater reliability for pass/fail validity of videos were calculated using the kappa statistic and percent agreement. All statistical analyses were performed using JMP 15.2 (SAS Institute, Cary, NC).

**Table 1:** Demographic and cognitive summary data of study participants at study start.

**Dog ID**	**Age** **(years)**	**Weight** **(kg)**	**Breed**	**Sex**	**Life stage**	**CADES score** **(average)**	**CADES Category**	**CI stage** **via CADES**
1	13.9	18	Shepherd Mix	FS	Geriatric	2	1	Normal
3	13.5	17	Terrier Mix	FS	Geriatric	7	1	
5	8	27.3	Plott Hound Lab Mix	MN	Senior	2	1	
7	11	21.9	Pitbull	FS	Senior	6	1	
8	9	24	Labrador Retriever Mix	FS	Senior	2	1	
9	10.5	8.4	Shetland Sheepdog Mix	MN	Senior	0	1	
10	10.5	32.7	Golden Retriever	MN	Geriatric	0	1	
11	13.5	8.2	Pembroke Welsh Corgi	FS	Geriatric	0	1	
12	13	16.3	Australian Shepherd	MN	Geriatric	6	1	
15	13.3	30.5	Shepherd Mix	MN	Geriatric	5	1	
18	14	7.2	Jack Russell Terrier Mix	MN	Geriatric	0	1	
19	9	34	Labrador Retriever	MN	Senior	0	1	
20	9.5	32	Bernese Mountain Dog	FS	Geriatric	3	1	
21	12.2	34	Golden Retriever	MN	Geriatric	2	1	
2	9.5	30	German Shepherd Mix	MN	Geriatric	9	2	Mild
4	10	9.1	Jack Russell Terrier Cross	MN	Senior	15	2	
6	7	54.5	Rottweiler	MN	Senior	14	2	
14	9.5	5.9	Dachshund	FS	Senior	19	2	
16	9.5	28.2	German Shepherd Mix	FS	Geriatric	9	2	
17	12	20.5	Border Collie	MN	Geriatric	13	2	
13	15.5	15	Jack Russell Terrier	MN	Geriatric	82	4	Severe

*FS, female spayed; MN, male neutered; CADES, CAnine DEmentia Scale; CI, cognitive impairment*.

## Results

### Participants

Twenty-three dogs met the inclusion criteria. Of these, two owners were unable to record and upload videos and were excluded. Twenty-one dog/owner pairs participated and details of the dogs are provided in Table 1, grouped according to CADES category.

### Sustained Gaze Test

Based on preliminary attempts and owner feedback at performing and videotaping the sustained gaze test, instructions were expanded to include more precise guidelines on when to start recording. It was common that dogs were already gazing at their owners at the start of the video and so the true start of the test could not be determined. Because so many dogs immediately fixated on their owners, owners were asked to start recording, then make a gesture away or some other movement to distract the dog, and then call the dog's name for the test to start. The maximum recording time was also expanded due to several owners ending the recording just prior to 60 s of gaze time and preventing a true end to the test. To prevent artificially shortened tests, instructions were modified to include the removal of potential distractors from the room, such as dog toys, bedding, or clothing, prior to testing. Instruction modifications to enhance video quality included adequate lighting in the room, especially with darker colored breeds, and keeping the dog's face in frame.

In total, 20 participants completed all 3 weeks of sustained gaze testing and 162 videos were submitted. One owner was lost to follow up and did not complete all 3 weeks of testing. Weekly email reminders were necessary for owners to successfully remain on schedule. Before beginning the study, 18 of the 21 participants (85.7%) chose to receive email reminders 1 day prior to completing their next sustained gaze test. Even with weekly email reminders, 8 of the 21 participants (38.1%) required multiple reminders for at least one of their testing sessions. With reminder emails, owners were successful at completing the sustained gaze testing and submitting videos on time. We received 82.7% submissions on time. On average, video submissions were made every 9 days (median = 8 days). Owners demonstrated they are willing and able to perform the sustained gaze test at home with their dogs.

### Video Analysis

Of the 183 videos submitted, 162 (88.5%) were acceptable and 21 were excluded. Videos were excluded if the rater could not see the entirety of the gaze or external distractions altered the dog's gaze. Specific external distractions which resulted in exclusion of videos included: owners repeating the dog's name, owners repeatedly stating a command (e.g., sit), other household pets entering the room during the test, other household members making loud noises, and subjects becoming distracted while performing an additional behavior (i.e., scratching, sneezing). The most common reasons for exclusion were owners not recording the entirety of the dog's gaze (42.9% of excluded videos) and owners repeatedly stating the dog's name or giving the dog commands (28.6% of excluded videos).

Many dogs exhibited behaviors during the test that did not necessarily prevent the test from being conducted successfully (Table 2). In 35.5% of submitted videos, dogs changed position or posture, with 70% of dogs exhibiting the behavior in at least one of their test videos. Videos were not excluded if the dog's gaze remained intact or the dog's intent to continue the test was clear. The second most common behavior, looking back and forth from the treat to the owner's face to the phone, was seen in 16.4% of submitted videos and 40% of the dogs. As long as the dog's face remained in the video frame and focused on the treat, phone, or owner, we did not exclude the video due to the behavior. The third most common behavior seen in submitted videos was barking by the dogs (14.2%). Overall, excitement, frustration, and anxiety-related behaviors (i.e., repeated position changes, barking, circling, encroaching on the owner's space (potentially causing difficulty maintaining video capture of the eyes), and jumping on the owner) accounted for the majority of the behaviors seen. Few of these behaviors negatively impacted video analysis resulting in exclusion of the video because the dogs maintained eye contact and the owners were able to keep the dog's face within the video frame.

**Table 2:** Summary of behaviors that occurred during the sustained gaze test.

**Behavior**	**# of dogs** **exhibiting (%)**	**# of videos** **(% of total)**
Scratching	1 (5%)	1 (0.5%)
Sneezing	1 (5%)	3 (1.6%)
Circling	1 (5%)	5 (2.7%)
Jumping on owner	2 (10%)	7 (3.8%)
Barking	3 (15%)	26 (14.2%)
Encroaching on owner's space	6 (30%)	20 (10.9%)
Eye movement from treat to camera	8 (40%)	30 (16.4%)
Changing positions/posture	14 (70%)	65 (35.5%)

### Intra-Individual Test-Retest Reliability of Gaze Duration and CADES

**Figure 2:**
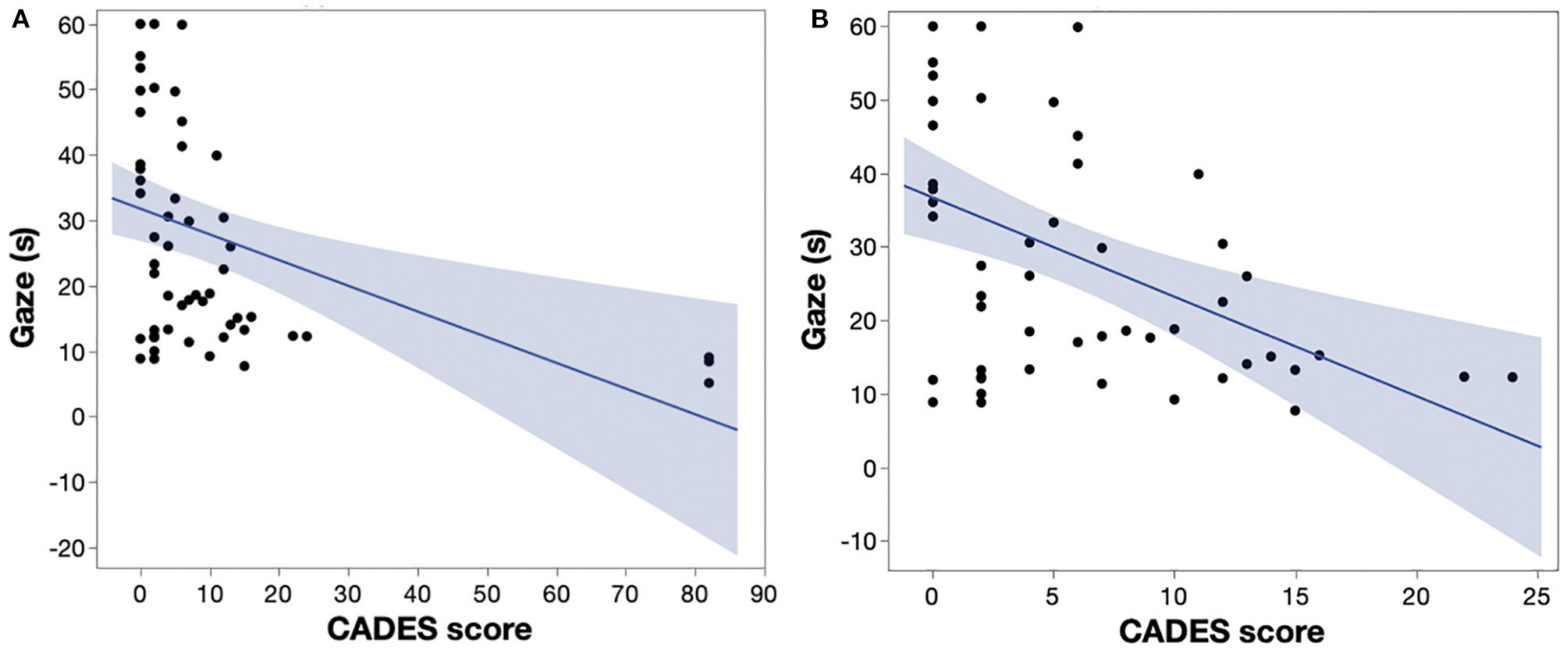
**(A)** There is a significant relationship between CADES score and sustained gaze duration (*r*^2^: 0.16, *p* = 0.0026). **(B)** When the outlier values from the severely affected dog are excluded, there is a significant relationship between CADES score and sustained gaze duration (*r*^2^: 0.21, *p* = 0.0005). CADES: CAnine DEmentia Scale; s: seconds.

There was a wide range of duration of sustained gaze test results with 5 dogs able to reach the 60 s limit. There was also a range of cognitive impairment as quantified by CADES, although the majority of dogs were classed as normal or mildly affected. Dogs were grouped according to CADES category and the summary data for their sustained gaze testing, breed, and age are provided in Table 1. A multivariate analysis was performed to examine the relationship between age, CADES score, and sustained gaze duration using logistic regression with age and CADES score as variables. There was a significant relationship between sustained gaze duration and CADES score (*p* = 0.0026) but not age (*p* = 0.65) (Figure 2A). Given the lack of dogs with moderate CADES score, the most severely affected dog (Dog ID#13) was excluded as an outlier and the analysis was repeated. There was still a significant relationship (*p* = 0.0005) between sustained gaze duration and CADES score (Figure 2B).

**Figure 3:**
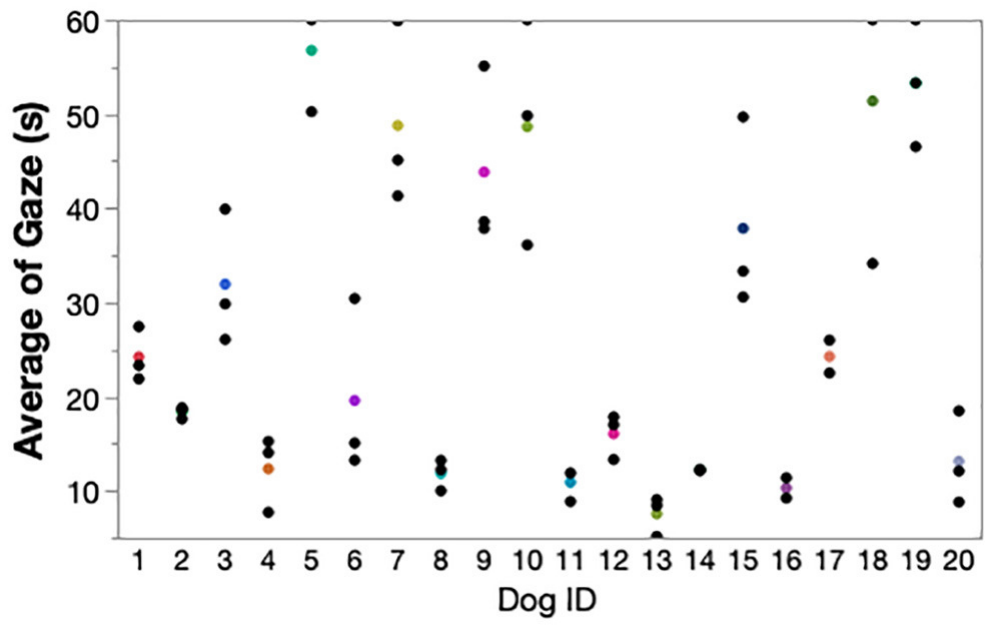
Test-retest reliability of the sustained gaze test performed at weekly intervals for 3 weeks. The colored points are the mean gaze duration for each dog and the black points are the mean gaze durations for each of the 3 week testing points. ICC: 0.85. ICC: Intraclass Correlation Coefficient; s: seconds.

When comparing the mean sustained gaze times over the 3 weekly testing sessions, the ICC was 0.85. An ICC of 0.8–1.00 is considered excellent and indicates high reliability between the tests performed by owners (Figure 3).

**Figure 4:**
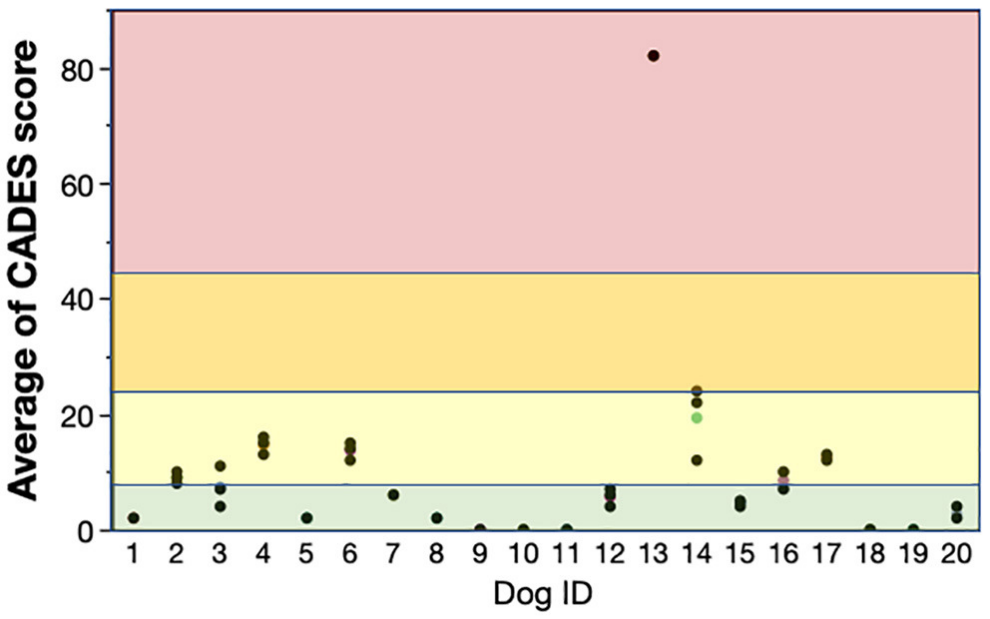
Test-retest reliability of the owner completed CADES score at weekly intervals for 3 weeks. The colored points are the mean CADES for each dog and the black points are the weekly score. The green background are scores that are considered normal, light yellow is mild dementia, darker yellow is moderate dementia and red is severe dementia. Score of dogs in the mild category did change a little week by week but this change rarely resulted in a change in dementia category. ICC: 0.99. CADES: CAnine DEmentia Scale; ICC: Intraclass Correlation Coefficient.

The test-retest reliability of CADES score showed high reliability across the 3 weeks of testing with an ICC of 0.99. The agreement of CADES category across the 3 weeks of testing was substantial with a Kappa statistic = 0.80 (*k* = 0.61–0.80, substantial agreement) (26). It was noteworthy that while 10 dogs did have changes in CADES score between testing weeks, in only three dogs did this change their category. The highest variability of CADES scores occurred for dogs in CADES category 2 (Figure 4).

### Intra- and Inter-rater Reliability of Gaze Times

The ICC of gaze duration measured by a single rater (JH) for valid videos only was extremely high at 0.99. The inter-rater reliability of timing valid videos was also excellent with an ICC of 0.96. The agreement both within and between raters on video pass/fail validity was substantial with a Kappa statistic = 0.76 (intra-rater) and 0.78 (inter-rater) (*k* = 0.61–0.80, substantial agreement) (26).

## Discussion

Our project demonstrated that owners are willing and able to successfully perform sustained gaze testing at home with their pet dogs. We easily recruited owners and quickly achieved our goal of 20 owner-dog combinations completing the full 3 weeks of testing. The ease in which dog-owner combinations meeting inclusion criteria were recruited demonstrates the potential feasibility of future large scale study recruitment.

In order for owners to accurately perform the sustained gaze test, pre-test video submissions were critical to ensuring both the quality and validity of owner submitted videos. We discovered owners need to be reminded to start recording their dog before they get their dog's attention, record past 60 s, and to not communicate verbally with their dog during the test. We modified our training module language to instruct owners to begin recording, then purposefully distract their dog, before finally redirecting the dog's attention to the owner's face/treat. Some suggestions offered to owners included: the owner tapping the floor with their foot, the owner dropping a piece of the treat on the floor, or the owner pretending to toss the treat. When dogs were excited and/or anxious during the sustained gaze test, many owners instinctively asked their dog to sit or stay, potentially artificially extending their dog's gaze duration. We recognized the need to modify our training module language to clearly articulate the dogs are allowed to change position and/or bark as long as they remained focused on the owner's face/treat.

Weekly email reminders were necessary to ensure timely owner sustained gaze test performance and video submission. Initially, it was thought repeated reminder emails would be antagonistic and off-putting for owners. However, the majority of owners requested weekly email reminders prior to starting the study. Owners voiced appreciation for the increased communication to ensure timely submissions and correct test performance, preventing late or repeated submissions. This is an important consideration when planning investigator time or number of animals to be included in a study.

We discovered unexpected behaviors, listed in Table 2, during the video analysis. Even with dogs performing the sustained gaze test on non-slip and comfortable surfaces, many dogs needed to reposition to remain comfortable. Since our study population only included senior and geriatric dogs, it was expected that some dogs may need to adjust their posture possibly from joint pain, muscle weakness, etc. Initially, owners were asked to stop recording after their dog broke eye contact, but we had to modify our instructions to account for repositioning. If repositioning occurred early in the video, causing the dog to briefly break their gaze while repositioning, we asked owners to continue recording with the second position as the start of the test. However, many dogs were able to reposition without breaking their gaze. With owners holding a treat in one hand and their phone in another, some dogs exhibited eye movement back and forth between the treat, the owner's face, and the phone. Since the phone and treat were in close proximity to the owner's face and the dogs remained engaged, we did not discard videos exhibiting this behavior. The eye movement seen with this behavior may exclude the use of video analysis software to measure gaze time durations.

Not surprisingly, many of the behaviors exhibited by the dogs could be due to anxiety, excitement, and/or frustration in anticipation of receiving a treat (27). If the dog did not break their gaze, we scored videos as valid. If the dog did break their gaze and had clear intent to continue the test, judgement on the validity of the video became more subjective. The circling behavior exhibited by one dog was the most challenging to score. Technically, the dog broke eye contact with every circle, but the dog's intent was clear in continuing the test. Due to excessive anxious and/or repetitive behaviors, some dogs may not be good candidates for the home sustained gaze test.

Our work demonstrated that owners can perform valid and reliable sustained gaze tests at home with their pet dogs. This supports and extends work showing comparable results for eye contact tests between populations of dogs tested at home vs. in a laboratory setting (23). We also demonstrated that owner submitted videos can be scored reliably by multiple raters with our exclusion criteria and scoring guidelines. Limitations of the sustained gaze test study design were revealed during the video analysis. The largest variation in the inter-rater reliability was due to the subjective nature of some observed behaviors, such as circling or barking. Due to the individualized quality of this portion of the video analysis and variability in dog populations, decisions regarding the validity of certain behaviors will depend on individual projects. However, external distractions of any kind (e.g., another pet entering the room or owners repeatedly stating the dog's name) were easily and consistently recognized by multiple raters. When videos were valid, multiple raters were extremely reliable in measuring gaze times.

Even though the initial aim of this project was to evaluate the validity and reliability of owner performed sustained gaze tests on pet dogs, we also evaluated the reliability of owner completed CAnine DEmentia Scale (CADES) questionnaires. The CADES questionnaire is designed to quantify the frequency of behavioral changes associated with CCDS and was developed for the veterinarian to complete *via* owner interviews and not completion by the owner (2). In our study, owners completed the CADES questionnaire weekly in conjunction with their video submissions. While our data showed both an extremely high reliability of CADES scores and substantial agreement of CADES categories across the 3 weeks of testing, we discovered some variability of CADES scores among dogs in CADES category 2 (mild cognitive impairment). Owners are confident when their dog is cognitively normal, but once mild changes occur, owners have less confidence in the exact frequency of the changes they observe. Owners may experience more difficulty in quantifying behaviors seen in dogs experiencing mild cognitive changes due to increased variability in the dog's day to day behaviors. Further study is needed to evaluate the cause and the significance of the variability we observed. This variability notwithstanding, given the relationships shown between the CADES score, sustained gaze performed in the laboratory, and biomarkers of neuronal dysfunction (12, 14), the ability of owners to complete both the CADES and sustained gaze tests in their homes marks an advance in our ability to study CCDS in aging dogs.

Our project establishes that a reliable and valid cognitive test can be completed by owners at home with minimal training. We believe at home cognitive testing of pet dogs will facilitate recruitment to future canine aging projects. Duration of eye contact in dogs has previously been shown to decrease with age (with no data on CADES performance) (28) and may be affected by the amount of training dogs have undergone (29). Further research with a larger cohort of dogs is needed to further determine the relationship between all these variables and sustained gaze duration. It is notable that our project was limited by the lack of dogs with moderate and severe cognitive impairment in the study cohort, which is perhaps due to selection bias of our owner population. Owners of dogs with moderate to severe cognitive impairment might have had less interest in attempting to perform cognitive testing at home. In the future, we aim to deploy the sustained gaze test to a large-scale community project and recruit 1,000 dog-owner combinations, across a broad range of adult and senior dogs with varying CADES scores. Since owners would be able to perform sustained gaze testing at home, we can expand our study population several fold compared to the number of dogs that could feasibly test in a single laboratory setting. If results are substantiated in a larger trial, the ease of the sustained gaze test (which can be performed with minimal training and equipment) makes cognitive testing and earlier diagnosis of CCDS in dogs more accessible to general practitioners (10, 30). Additionally, expanding the implementation of the sustained gaze test may allow practitioners to estimate the prevalence of CCDS more accurately as current estimates of CCDS prevalence vary widely between studies (10, 12).

## Data Availability Statement

The raw data supporting the conclusions of this article will be made available by the authors, without undue reservation.

## Ethics Statement

The animal study was reviewed and approved by NCSU Institutional Animal Care and Use Committee. Written informed consent was obtained from the owners for the participation of their animals in this study.

## Author Contributions

NJO conceived and designed the study and created the sustained gaze training video. JH recruited and trained participants, created the video uploading training video, and conducted video analysis. MG contributed to study design and video analysis methodology. JH and NJO wrote the manuscript. GT, GF, BC, AS, and MG provided critical feedback. GF, BC, AS, MG, and NJO conducted video analysis testing. All authors contributed to the article and approved the submitted version.

## Funding

JH was funded by NIH Interdisciplinary Biomedical Research Training Program T35-T35OD011070.

## Conflict of Interest

The authors declare that the research was conducted in the absence of any commercial or financial relationships that could be construed as a potential conflict of interest.

## Publisher's Note

All claims expressed in this article are solely those of the authors and do not necessarily represent those of their affiliated organizations, or those of the publisher, the editors and the reviewers. Any product that may be evaluated in this article, or claim that may be made by its manufacturer, is not guaranteed or endorsed by the publisher.
