# Supplementary material for: Sustained Gaze Is a Reliable In-home Test of Attention for Aging Pet Dogs
Source: Front Vet Sci. 2021 Dec 23;8:819135. doi: 10.3389/fvets.2021.819135 (PMC8732866; doi:10.3389/fvets.2021.819135)
Supplement: Supplementary file 1 [file Data_Sheet_1.PDF]

The Sustained Gaze Study Questionnaire

This is a series of questions about your pet and your pet's cognition

1. Please enter your email address

Please answer the following questions, review the training module (a link is provided after the questions) and then upload your 3 videos of your dog's face during the sustained gaze test. THANK YOU.

2. What is your first and last name?
3. What is your address (street, city, and state)?
4. What is your dog's name?
5. What is your dog's breed?
6. What is your dog's date of birth? If you don't know their date of birth, you can enter their approximate age.
7. What is your dog's sex?
    a. Spayed female
    b. Female
    c. Neutered male
    d. Male
8. What is your dog's weight? Please note if pounds or kg.

The following questions together allow us to assign a score to your dog's level of cognitive dysfunction in its normal daily life. Together these questions form the CADES questionnaire.

9. How often is your dog disoriented in a familiar environment (inside/outside)?
    a. Never
    b. Once in the last 6 months
    c. Once per month
    d. 2-4 times per month
    e. Several times a week
10. How often does your dog have difficulties recognizing familiar people and animals inside or outside your home?
    a. Never
    b. Once in the last 6 months
    c. Once per month
    d. 2-4 times per month
    e. Several times a week
11. How often does your dog respond abnormally to familiar objects (e.g. a chair, a trash can)?
    a. Never
    b. Once in the last 6 months
    c. Once per month
    d. 2-4 times per month
    e. Several times a week

12. How often does your dog aimlessly wander during the day?
    a. Never
    b. Once in the last 6 months
    c. Once per month
    d. 2-4 times per month
    e. Several times a week
13. How often does your dog show a reduced ability to do a previously learned task?
    a. Never
    b. Once in the last 6 months
    c. Once per month
    d. 2-4 times per month
    e. Several times a week
14. How often do you find changes in your dog's interaction with people and other dogs (playing, petting, welcoming)?
    a. Never
    b. Once in the last 6 months
    c. Once per month
    d. 2-4 times per month
    e. Several times a week
15. How often do you see changes in individual behaviors of your dog (exploratory behavior, play, performance)?
    a. Never
    b. Once in the last 6 months
    c. Once per month
    d. 2-4 times per month
    e. Several times a week
16. How often does your dog exhibit a reduced response to commands and ability to learn new tasks?
    a. Never
    b. Once in the last 6 months
    c. Once per month
    d. 2-4 times per month
    e. Several times a week
17. How often do you find your dog getting irritable?
    a. Never
    b. Once in the last 6 months
    c. Once per month
    d. 2-4 times per month
    e. Several times a week
18. How often does your dog express aggression?
    a. Never
    b. Once in the last 6 months
    c. Once per month
    d. 2-4 times per month
    e. Several times a week

19. How often does your dog have abnormal behavior at night – waking up, wandering, vocalizing?
    a. Never
    b. Once in the last 6 months
    c. Once per month
    d. 2-4 times per month
    e. Several times a week
20. How often does your dog switch between inability to sleep and excessive sleeping?
    a. Never
    b. Once in the last 6 months
    c. Once per month
    d. 2-4 times per month
    e. Several times a week
21. How often does your dog eliminate at home in random locations?
    a. Never
    b. Once in the last 6 months
    c. Once per month
    d. 2-4 times per month
    e. Several times a week
22. How often does your dog eliminate at home in its kennel or sleeping area?
    a. Never
    b. Once in the last 6 months
    c. Once per month
    d. 2-4 times per month
    e. Several times a week
23. How often does your dog exhibit changes in signaling the need to eliminate?
    a. Never
    b. Once in the last 6 months
    c. Once per month
    d. 2-4 times per month
    e. Several times a week
24. How often does your dog eliminate indoors after a recent walk outside?
    a. Never
    b. Once in the last 6 months
    c. Once per month
    d. 2-4 times per month
    e. Several times a week
25. How often does your dog eliminate in uncommon locations such as on concrete?
    a. Never
    b. Once in the last 6 months
    c. Once per month
    d. 2-4 times per month
    e. Several times a week

We have provided a short training module on how to do this test. It can be found at this link: https://youtu.be/yMFUtQ-9qz0

Please upload 3 videos of your dog's face as you do the sustained gaze test. Please contact us if you have any issues uploading your videos.

26. What time of day did you record the videos you uploaded?
    a. Early Morning (5am-8am)
    b. Morning (8am-11am)
    c. Midday (11am-2pm)
    d. Afternoon (2pm-5pm)
    e. Early Evening (5pm-8pm)
    f. Late Evening (8pm-11pm)
    g. Overnight (11pm-5am)
27. Please describe any events that might have distracted your dog if you feel they are relevant
28. Please provide any comments you have about performing this test with your dog in the space below. Thank you.
